# A Long Non-coding RNA IVRPIE Promotes Host Antiviral Immune Responses Through Regulating Interferon β1 and ISG Expression

**DOI:** 10.3389/fmicb.2020.00260

**Published:** 2020-02-20

**Authors:** Lingna Zhao, Min Xia, Keyu Wang, Chengcai Lai, Hongxia Fan, Hongjing Gu, Penghui Yang, Xiliang Wang

**Affiliations:** ^1^State Key Laboratory of Pathogen and Biosecurity, Beijing Institute of Microbiology and Epidemiology, Beijing, China; ^2^The Fifth Medical Center of Chinese PLA General Hospital, Beijing, China

**Keywords:** lncRNA – long non-coding RNA, IFNβ1, IAV, ISGs, IVRPIE

## Abstract

Accumulating studies have shown that long non-coding RNAs (lncRNAs) modulate multiple biological processes, including immune response. However, the underlying mechanisms of lncRNAs regulating host antiviral immune response are not well elucidated. In this study, we report that analysis of the existing dataset transcriptome of blood immune cells of patients with influenza A virus (IAV) infection and after recovery (GSE108807) identified a novel lncRNA, termed as IVRPIE (Inhibiting IAV Replication by Promoting IFN and ISGs Expression), was involved in antiviral innate immunity. *In vitro* studies showed that IVRPIE was significantly upregulated in A549 cells after IAV infection. Gain-and-loss of function experiments displayed that enforced IVRPIE expression significantly inhibited IAV replication in A549 cells. Conversely, silencing IVRPIE promoted IAV replication. Furthermore, IVRPIE positively regulates the transcription of interferon β1 and several critical interferon-stimulated genes (ISGs), including IRF1, IFIT1, IFIT3, Mx1, ISG15, and IFI44L, by affecting histone modification of these genes. In addition, hnRNP U was identified as an interaction partner for IVRPIE. Taken together, our findings suggested that a novel lncRNA IVRPIE is a critical regulator of host antiviral response.

## Introduction

Influenza A virus (IAV) infection poses great challenges to the public health (Neumann et al., 2009). Numerous researches attempted to explore the mechanisms of host antiviral immune responses. The innate immune system builds the first line defense against IAV infection. The initial sensing of IAV infection is mediated by innate pattern recognition receptors (PRRs), such as retinoic acid-inducible gene I (RIG-I), melanoma differentiation factor 5 (MDA5), and toll-like receptor 3 (TLR3) (Alexopoulou et al., 2001; Kato et al., 2005; Gitlin et al., 2006; Takeuchi and Akira, 2010). After sensing IAV infection, they signal through different adaptor proteins, MAVS or TRIF, to activate two cytosolic protein kinase complexes, one consisting of IKKα, IKKβ and NEMO, and the other comprising TBK1 (TANK-binding kinase 1) or IKK-i/ε. The IKKα, IKKβ and NEMO complex subsequently free NF-κB to translocate into the nucleus and activate expression of proinflammatory cytokine genes. And the TBK1 complex leads to phosphorylation and dimerization of the transcription factors IRF3 and IRF7, which translocate to the nucleus, triggering a rapid production of type I IFN genes (Kawai et al., 2005; Seth et al., 2005; Takeuchi and Akira, 2010). Then type I IFN stimulates cells to initiates a signaling cascade that leads to phosphorylation and heterodimerization of STAT1 and STAT2, which interact with IRF9 to form ISGF3, regulating the synthesis of many IFN-stimulated genes (ISGs), including IRF1, IFIT1, IFIT3, Mx1, ISG15, and IFI44L, serving as an essential primary barrier for virus infection (Darnell et al., 1994; Kotenko et al., 2003; Stark and Darnell, 2012; Au-Yeung et al., 2013).

Although cell signaling pathways of host antiviral immune responses have been well understood, the majority of studies have focused on the function of proteins, and less is known about the roles of RNA in this process. In the past two decades, studies have demonstrated that thousands of non-coding RNAs are universally transcribed from the Genome, one of which is classified as long non-coding RNAs (lncRNAs) (Kapranov et al., 2002, 2007; Birney et al., 2007; Djebali et al., 2012). LncRNAs are generally defined as transcripts longer than 200 nucleotides that lack protein-coding potential (Derrien et al., 2012; Djebali et al., 2012). Accumulating data indicate that lncRNAs play essential roles in nearly every biological process, including transcription, splicing, translation, imprinting, cell “stemness,” differentiation, adaptation and death (Mercer et al., 2009; Johnsson et al., 2014). Recent studies have found that lncRNAs also have important roles in host immune response (Atianand and Fitzgerald, 2014; Chen et al., 2017). In viral infections, several lncRNAs were reported to regulate viral replication. For example, 7SL and NEAT1 are involved in modulating HIV-1 posttranscriptional expression (Wang et al., 2007; Zhang et al., 2013). NRAV and lnc-ISG20 were found to affect IAV replication and virulence (Ouyang et al., 2014; Chai et al., 2018). CCR5AS can diminish infection of CD4^+^ T cells with HIV (Kulkarni et al., 2019). However, the vast majority of lncRNAs during IAV replication remain uncharacterized.

In this study, we defined a novel human lncRNA, termed as IVRPIE, as a critical regulator in anti-IAV infection. A549 cells with IAV infection upregulated IVRPIE, compared with control cells. *In vitro* data revealed that IVRPIE served the function of antiviral activity by promoting IFNβ1 and several ISG production. Furthermore, we found that IVRPIE promoted the expression of these genes through affecting chromatin remodeling at their transcription starting site by interaction with heterogeneous nuclear ribonuclear protein U (hnRNP U). These results demonstrate that IVRPIE is a positive regulator of IFNβ1 and ISG expression, establishing a critical role in host innate defense during the IAV infection.

## Materials and Methods

### Cell Lines and Virus Strains

Human lung adenocarcinoma epithelial cells (A549) and human bronchial epithelium BEAS-2B cells were grown in F12 supplemented with 10% (vol/vol) FBS (Royacel) and antibiotics (penicillin and streptomycin) (Invitrogen) at 37°C under 5% CO_2_ concentration. Madin-Darby canine kidney (MDCK) cells and BabyHamster Syrian Kidney (BHK21) cells were grown in DMEM supplemented with 10% (vol/vol) FBS (Royacel) and antibiotics (penicillin and streptomycin) (Invitrogen).

A/Beijing/501/2009 (H1N1; BJ501), A/Puerto Rico/8/34 (H1N1; PR8), A/Singapore/INFIMH-16-0019/2016 (H3N2; SI16) and Sendai virus (SeV) were propagated in embryonated chicken eggs. VSV New Jersey (VSNJV) and VSV Indiana (VSIV) were propagated in BHK21 cells. Respiratory Syncytial Virus (RSV) was propagated in Hep2 cells. Adenovirus was propagated in Vero cells.

All of the experimental protocols used in this study were approved by the Institutional Animal Care and Use Committees of the Beijing Institute of Microbiology and Epidemiology (permit number: SYXK2015-008). And all of the experiments were performed in strict accordance with the approved guidelines.

### Antibodies and Reagents

The antibodies used were IRF-1 (8478S, Cell Signaling), IFIT1 (14769S, Cell Signaling), IFIT3 (sc-393512, Santa Cruz Biotechnology), Mx1 (37849S, Cell Signaling), ISG15 (2758T, Cell Signaling), IFI44L (HK7931, Hushi Pharmaceutical Technology), Tri-Methyl-Histone H3 (Lys27) (C36B11) Rabbit mAb (9733, Cell Signaling), Tri-Methyl-Histone H3 (Lys4) (C42D8) Rabbit mAb (9751, Cell Signaling), anti-rabbit IgG, HRP-linked antibody (7074P2, Cell Signaling), anti-mouse IgG, HRP-linked antibody (7076P2, Cell Signaling). Anti-A/California/7/2009-like HA serum (sheep 606 and 610) (14/310) was purchased from National Institute for Biological Standards and Control (NIBSC). Anti-sheep IgG, HRP-linked antibody (F030231) was purchased from Beijing BioRab Technology. Mouse anti-hnRNP U monoclonal antibody (ab10297) was purchased from Abcam.

Polyinosinic-polycytidylic acid sodium salt (P9582) was purchased from Sigma-Aldrich. Human IFN-β (10704-H02H) was purchased from Sino Biological. MRT67307 HCl (T5162) and Pyrrolidinedithiocarbamate ammonium (T3147) were purchased from TargetMol.

### Plasmids, siRNAs, and ASOs

The IVRPIE gene was cloned into the pcDNA 3.1(+) plasmid. Primers used in RT-PCR were listed in Supplementary Table S1.

IVRPIE-specific Antisense oligonucleotides (ASOs), and negative control ASO were synthesized by Guangzhou RiboBio Co., Ltd. (RiboBio). The targeting sequences of the ASOs were as follows: ASO1: GCACTACACTCTTGGCATAT; ASO2: ACCTATCCCGTGCTGTAAAT. hnRNP U-specific siRNAs and negative control siRNAs were synthesized by GenePharma Ltd. The targeting sequence of the hnRNP U-specific siRNA was as follows: hnRNP U-2443: CGUGGUAGUUACUCAAACATT, negative control: UUCUCCGAACGUGUCACGUTT. ASOs and siRNAs were transfected into A549 cells using Lipofectamine 3000 (Invitrogen) according to the manufacturer’s instructions.

### Bioinformatics Analysis of Non-coding Potential

Non-coding potential of IVRPIE was analyzed by coding potential calculator 2^1^ (Kang et al., 2017).

### Quantitative PCR

RNA was extracted from A549 cells using TRIzol Reagent (Invitrogen) according to the manufacturer’s instructions. cDNA was synthesized from total RNA using TransScript First-Strand cDNA Synthesis SuperMix (TransGen Biotech) and subjected to quantitative PCR using UltraSYBR Mixture (Low ROX) (CW2601M, ComWin Biotech) performed on QuantStudio 6 Flex system (ABI). Primers used in quantitative PCR were listed in Supplementary Table S1. The relative amounts of the tested RNAs were calculated using the 2^–ΔΔCt^ method against GAPDH.

### Rapid Amplification of cDNA Ends (RACE)

RACE of IVRPIE was performed using a SMARTer^TM^ RACE cDNA Amplification Kit (Clontech) according to the manufacturer’s instructions. The primers for IVRPIE 5′ RACE were as follows: IVRPIE outer R, 5′-TCTGTGGTGCACATGC TGTCTTCCGT-3′ and IVRPIE inner R, 5′-GATTACGCC AAGCTTAAGCATTTCCCGAGTTTGCTGGAACACA-3′. The primers for IVRPIE 3′ RACE were as follows: IVRPIE outer F, 5′-GATGAGAAGTCTGAGGCTTTACCTAAACTTCA-3′ and IVRPIE inner F, 5′- AAACTTATGATTAAAGGCTTCTA CGTACTCA-3′.

### Plaque Forming Assay (PFU), HA Assay and 50% Tissue Culture Infectious Dose (TCID_50_) Assay

For plaque forming assay, MDCK cells were seeded in 6-well plates and infected with serial dilutions of virus in serum-free DMEM for 1 h. Then, the cells were supplemented with DMEM containing 1% agarose (Promega) and 2 μg/mL of TPCK-trypsin. After the agar solidified at 4°C, plates were incubated upside-down at 37°C for 5 days. Then the virus plaques were stained with 1% crystal violet and counted, and PFUs of the virus were determined. For HA assay, the supernatants of cell culture were diluted with physiological saline and mixed with an equal volume of 1% chicken erythrocytes. The viral titers were counted from the highest dilution factors that produced a positive reading (Wang et al., 2012). For TCID_50_ assay, BHK21 cells were seeded in 96-well cell culture plates and infected with serial 10-fold dilutions of VSNJV made in DMEM medium containing 2% (vol/vol) FBS for 48 h. Then cytopathic effect (CPE) of each of the wells of the plate was examined and TCID_50_ was calculated by Reed-Muench method.

### Western Blotting

Cells were washed once by ice-cold PBS, and then were lysed using RIPA buffer (R0010, Solarbio) containing Phenylmethanesulfonyl fluoride (PMSF) (P0100, Solarbio). Proteins were separated by SDS-PAGE, and then transferred onto PVDF membrane (Roche Diagnostics). PVDF membranes were incubated with the following diluted primary antibodies: Anti-A/California/7/2009-like HA serum (sheep 606 and 610) (14/310, NIBSC), IRF-1 (8478S, Cell Signaling), IFIT1 (14769S, Cell Signaling), IFIT3 (sc-393512, Santa Cruz Biotechnology), Mx1 (37849S, Cell Signaling), ISG15 (2758T, Cell Signaling), IFI44L (HK7931, Hushi Pharmaceutical Technology). Membranes were washed three times in Tris-buffered saline containing 1% Triton X-100 and then were incubated with horseradish peroxidase (HRP)-conjugated secondary antibodies (7074P2, Cell Signaling, F030231, Beijing BioRab Technology). Immunoreactive bands were visualized by enhanced chemiluminescence using Super ECL Plus Detection Reagent (P1050, Applygen) using Tanon-5200 (Tanon).

### Subcellular Fractionation

Cytoplasmic and nuclear fractions were separated using PARIS Kit Protein and RNA Isolation System (Invitrogen) according to the manufacturer’s instructions. Briefly, A549 cells were lysed with Cell Fractionation Buffer on ice for 5–10 min. The lysates were centrifuged 1–5 min at 4°C and 500 × *g*. The supernatant cytoplasmic fraction was aspirated away from the nuclear pellet and transferred to a new tube. The nuclear pellets were washed with Cell Fractionation Buffer once and lysed with Cell Disruption Buffer by vigorous vortex or pipet. RNA was extracted using TRIzol Reagent and quantitative PCR was performed to detect IVRPIE expression in cytoplasmic and nuclear fraction.

### RNA Pull-Down Assay and Mass Spectrometry

RNA pull-down assay was carried out using Magnetic RNA-Protein Pull-Down Kit (Thermo Scientific Pierce). Label the target RNA using the included Thermo Scientific Pierce RNA 3′ Desthiobiotinylation Kit. 25–100 pmol of labeled RNA was bound to Streptavidin Magnetic Beads. Add 400 μL of Master Mix containing A549 nuclear lysate to the RNA-bound beads. Add 50 μL of Elution Buffer to the beads and mix well by vortexing. The eluted proteins were subjected to silver-staining (Invitrogen Silver Staining Kit, Thermo) and whole bands were excised and sent for LC-MS/MS analysis which was performed by Shanghai Luming Biological Technology Co., Ltd.

### RNA Immunoprecipitation (RIP)

hnRNP U RIP experiments were performed in nuclear extracts isolated from unstimulated A549 cells under native conditions. Assays were performed as previously described (Tsai et al., 2010). Nuclear extracts were immunoprecipitated with 2.5 μg hnRNP U (Abcam, clone 4D11, ab6106) and isotype-matched control IgG antibodies overnight. RNA-protein-antibody complexes were captured using Dynabead Protein A/G (Thermo Fisher Scientific). RNA was eluted by adding TRIzol directly to magnetic beads and isolated as per manufacturer’s instructions. cDNA was synthesized using TransScript First-Strand cDNA Synthesis SuperMix (TransGen Biotech) and analyzed by qPCR. Results were normalized to input RNA and shown as fold-enrichment over control IgG RIP.

### Chromatin Immunoprecipitation (ChIP)

A549 cells overexpressing or knocking down IVRPIE were infected with BJ501 (MOI = 1, 24 hpi) and subjected to ChIP assays using the SimpleChIP^®^ Enzymatic Chromatin IP Kit (Magnetic Beads) (Cell Signaling Technology) following the manufacturer’s instruction. Briefly, 4 × 10^6^ cells were fixed and lysed in 1× Buffer A per immunoprecipitation. Nucleus fraction was pelleted and resuspended in 100 μL 1× Buffer B, followed by enzymic digestion of DNA by 0.5 μL of Micrococcal Nuclease for 20 min at 37°C to the length of approximately 150–900 bp and sonication to break nuclear membrane. Sheared chromatin was immunoprecipitated with 5 μL anti-H3K4me3 (CST; 9751), anti-H3K27me3 (CST; 9733) or Normal Rabbit IgG (CST) antibody at 4°C for 4 h to overnight followed by incubation with 30 μL ChIP-Grade Protein G Magnetic Beads for 2 h at 4°C with rotation. To reverse the cross-links, protein digestion with proteinase K and 5 M NaCl was performed. Immunoprecipitated DNA was purified and quantified by qPCR analysis using UltraSYBR Mixture (Low ROX) (CW2601M, ComWin Biotech). Primers used in qPCR were listed in Supplementary Table S1. Modificated histone enrichment in the ChIP samples were normalized to the input DNA. Experiments were performed at least three times with independent chromatin samples.

### Statistical Analysis

Statistical analyses were performed using GraphPad Prism version 5.0 (GraphPad Software Inc.). Student’s *t*-test was used to analyze the significance of data. *P* < 0.05 was considered statistically significant. Error bars represent standard error (± SEM).

## Results

### LncRNA IVRPIE Is Preferentially Up-Regulated in Patients With IAV Infection

**FIGURE 1:**
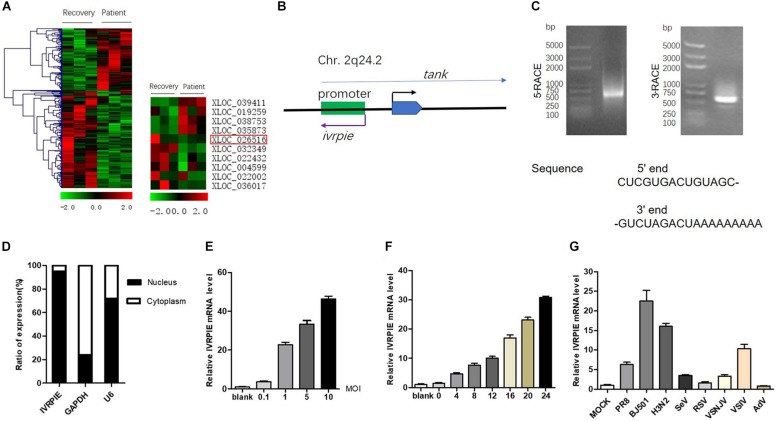
Identification and characterization of IVRPIE. **(A)** Analysis of existing dataset (GSE108807) of RNA sequencing of patients with IAV infection and after recovery revealed 118 upregulated and 187 downregulated lncRNAs (left). Ten lncRNAs are selected and shown in heatmaps (right). **(B)** Schematic diagram of IVRPIE and *tank*. **(C)** The 5′ and 3′ end sequences of IVRPIE determined by RACE. **(D)** Cytoplasmic and nuclear fractions from A549 cells were separated, and the RNA levels of IVRPIE in different subcellular fractions were determined by RT-qPCR. GAPDH mRNA and U6 RNA were respectively used as cytoplasmic and nuclear control. Data are shown as % input (means ± SD; *n* = 3). **(E,F)** A549 cells were infected with BJ501 at the indicated MOI for 24 h **(E)** or at 1 MOI for indicated hours **(F)**, and RT-qPCR was performed to determine the IVRPIE expression. **(G)** A549 cells were infected with different viruses, and RT-qPCR was performed to determine the IVRPIE expression. Data were normalized to GAPDH. Data are shown as the mean ± SD; *n* = 3.

To define lncRNAs involved in IAV infection, we analyzed the existing dataset (GSE108807) which utilized RNA sequencing to define the transcriptome of peripheral blood leucocyte samples from patients infected with IAV in the acute stage which were confirmed by molecular diagnostics and their matched recovery-stage (9 months after recovery), to depict the lncRNA profiles involved in IAV infection (Lai et al., 2019). As shown in Figure 1A and Supplementary Table S2, several lncRNAs that were differentially expressed in patients with IAV infection were chosen for function studies after confirmation by RT-qPCR (Supplementary Figure S1A).

To identify lncRNAs affecting IAV infection, virus titers were detected by HA assay after transient transfection of lncRNAs in A549 cells (Supplementary Figure S1B). IVRPIE was found to inhibit IAV replication most significantly, and thus it was selected for further study. The human lncRNA gene *virpie* (XLOC_026516) is located on chromosome 2q24.2, transcribed from the antisense strand of promotor region of *tank* locus (Figure 1B). Protein-coding potential analysis was performed by coding potential calculator 2^2^, and the low coding potential score (−1.0565) suggested that it’s a non-coding transcript (Kang et al., 2017) (Supplementary Figure S1C). 5′ and 3′ RACE studies were performed to define the exact length and sequence of IVRPIE. IVRPIE is exactly 1316 nt (Supplementary Table S3), and the 5′ and 3′ sequences are shown in Figure 1C. And IVRPIE is most located in nucleus (Figure 1D).

Furthermore, we observed that IVRPIE was upregulated in a virus dose- and infection time-dependent manner in A549 cells (Figures 1E,F). Besides, IVRPIE was significantly upregulated by some other RNA virus infections, including Sendai virus (SeV), Vesicular Stomatitis Virus (VSV) (Figure 1G), and Poly (I:C) (Supplementary Figure S1D), but not upregulated by some RNA viruses, like RSV, DNA viruses, like Adenovirus (AdV) (Figure 1G) and IFNβ (Supplementary Figure S1E). Similar results were obtained from another normal human bronchial epithelium cells, BEAS-2B cells (Supplementary Figures S1F,G). Surprisingly, except for A549 cells and BEAS-2B cells, IVRPIE was not significantly upregulated in other human cell lines (Supplementary Figure S1H), which revealed that it mainly exerted its function in blood immune cells and lung cells. To explore the regulatory mechanisms involved in IVRPIE expression, we evaluated the role of TBK1 and NF-κB in controlling the expression of IVRPIE. A549 cells were pretreated with DMSO, MRT67307 HCl, a kinase inhibitor of TBK1 and IKKε, or Pyrrolidinedithiocarbamate ammonium, a selective NF-κB inhibitor, followed by BJ501 infection for 24 h. Interestingly, upregulation of IVRPIE was not affected in MRT67307 HCl or Pyrrolidinedithiocarbamate ammonium-treated A549 cells relative to the control cells (Supplementary Figure S1I). These results indicate that IVRPIE expression is not regulated by TBK1 and NF-κB, which needs to be further studied. Together, these experiments demonstrate that upregulation of IVRPIE is mainly associated with some RNA viral infections, especially IAV virus.

### Upregulation of IVRPIE Inhibits IAV Replication *in vitro*

**FIGURE 2:**
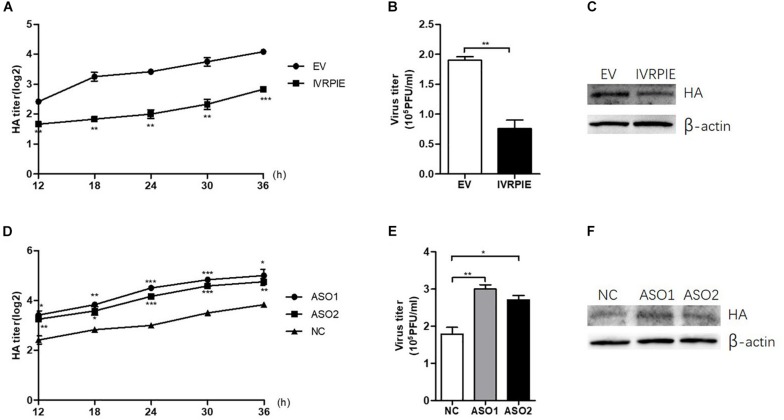
IVRPIE regulates IAV replication in A549 cells. **(A–C)** IVRPIE were transiently overexpressed in A549 cells, and IAV titers were respectively determined by HA assay **(A)**, PFU assay **(B)**, and western blotting **(C)**. **(D–F)** IVRPIE was knocked down by specific ASOs in A549 cells, and IAV titers were respectively determined by HA assay **(D)**, PFU assay **(E)**, and western blotting **(F)**. Data are shown as the mean ± SD; *n* = 3. *P < 0.05; **P < 0.01; ***P < 0.001 (Student’s *t*-test).

To study the effect of IVRPIE on viral load, an *in vitro* infection assay was performed in which A549 cells were transfected with IVRPIE or specific Anti-sense oligos (ASO) targeting IVRPIE. For IVRPIE overexpression, A549 cells were transfected with IVRPIE and then infected with IAV (BJ501) (Supplementary Figures S2A,B). The virus growth kinetics results showed that viral titers were lower in IVRPIE-overexpressing cells than control cells (Figures 2A,B). We further confirmed inhibition of IAV replication in IVRPIE-overexpressing cells by western blotting using an antibody against the IAV hemagglutinin (HA) (Figure 2C). Next, A549 cells were transfected with IVRPIE specific ASOs to downregulate IVRPIE (Supplementary Figure S2C), and the effect of IVRPIE downregulation on IAV viral load was analyzed as above. Virus titers were significantly higher in IVRPIE-reduced cells compared to control cells (Figures 2D–F). Similar results were obtained from IVRPIE-overexpressing and knockdown BEAS-2B cells (Supplementary Figures S2D,E). Besides, IVRPIE overexpression can inhibit the replication of other RNA viruses, such an VSV (Supplementary Figure S2F), while IVRPIE knockdown can promote the replication of VSV (Supplementary Figure S2G), determined by TCID_50_ assay. These results suggest that upregulation of IVRPIE in infected cells might be a host self defense mechanism to virus infection.

### IVRPIE Positively Regulates the Expression of IFNβ1 and Several Critical ISGs

**FIGURE 3:**
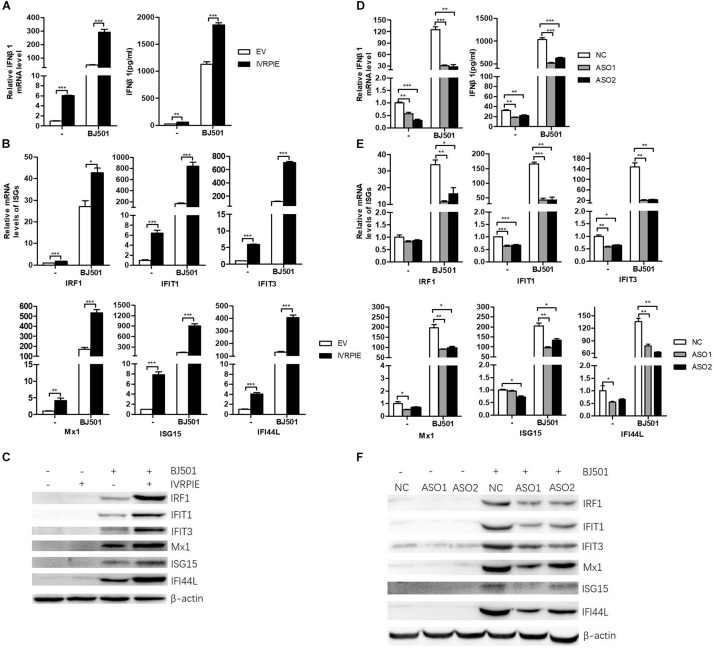
Altering IVRPIE expression in A549 cells changes the expression of IFNβ1 and several critical ISGs. **(A)** IVRPIE was transiently overexpressed in A549 cells, and mRNA level and protein level of IFNβ1 were detected by RT-qPCR and ELISA respectively. **(B,C)** mRNA levelsand protein levels of several critical ISGs were detected by RT-qPCR **(B)** and western blotting **(C)**. **(D)** IVRPIE was knocked down by specific ASOs in A549 cells, and mRNA level and protein level of IFNβ1 were detected by RT-qPCR and ELISA respectively. **(E,F)** mRNA levels and protein levels of several critical ISGs were detected by RT-qPCR **(E)** and western blotting **(F)**. For RT-qPCR, data were normalized to GAPDH. Data are shown as the mean ± SD; *n* = 3. *P < 0.05; **P < 0.01; ***P < 0.001 (Student’s *t*-test).

To define the mechanism by which IVRPIE inhibited IAV replication, we tested the IFN and ISGs mRNA and protein level by RT-qPCR, ELISA, or western blotting after overexpressing or knocking down IVRPIE in A549 cells. Notably, mRNA and protein levels of IFNβ1 and some critical ISGs were significantly upregulated in IVRPIE-overexpressing cells, including IRF1, IFIT1, IFIT3, Mx1, ISG15, and IFI44L (Figures 3A–C). Conversely, the mRNA and protein levels of IFNβ1 and these ISGs were reduced in IVRPIE knockdown cells compared to control cells (Figures 3D–F). Collectively, these results indicate that IVRPIE exerts antiviral functions by upregulating IFNβ1 and some critical ISGs.

### IVRPIE Was Involved in Regulation of Histone Modifications of IFNβ1 and ISGs

**FIGURE 4:**
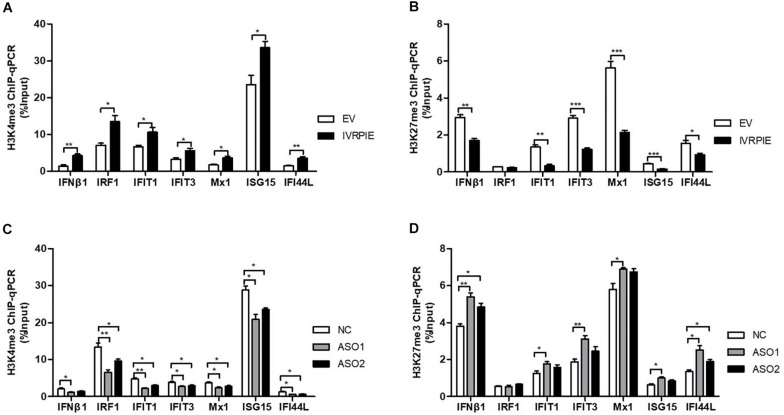
IVRPIE regulates the expression of IFNβ1 and ISGs through histone modifications of these genes. **(A,B)** ChIP-qPCR analysis of H3K4me3 **(A)** and H3K27me3 **(B)** at IFNβ1 and ISGs in IVRPIE overexpressing A549 cells that were infected with BJ501 for 24 h. **(C,D)** ChIP-qPCR analysis of H3K4me3 **(C)** and H3K27me3 **(D)** at IFNβ1 and ISGs in IVRPIE knock-down A549 cells that were infected with BJ501 for 24 h. DNA immuno precipitated by the anti-H3K4me3 antibody or anti-H3K27me3 antibody was calculated using the Percent Input Method. Data are shown as the mean ± SD; *n* = 3. *P < 0.05; **P < 0.01; ***P < 0.001 (Student’s *t*-test).

Next, we explored the mechanism involved in IVRPIE regulation of IFNβ1 and ISG expression. As IVRPIE is located in the nucleus, we investigated the relationship between IVRPIE and its adjacent gene, TRAF family member associated NFκB activator (TANK). It has been reported that TANK is involved in antiviral activities (Chariot et al., 2002; Lee et al., 2013), so we examined TANK expression levels after altering IVRPIE expression. Importantly, there was not significant change after overexpression or knocking down IVRPIE (Supplementary Figures S3A,B). These results demonstrated that other mechanisms might be involved in IVRPIE regulation of IFNβ1 and ISG expression. It was reported that lncRNAs can modulate the chromatin state at the transcription start site (TSS) of protein coding genes, so we investigated whether IVRPIE regulated IFNβ1 and ISG expression through this mechanism. We tested the histone modification at transcription start sites of IFNβ1 and ISGs by performing ChIP-qPCR in which histone 3 lysine 4 trimethylation (H3K4me3) and histone 3 lysine 27 trimethylation (H3K27me3) were, respectively, selected as an active mark and repressive mark of transcription. Notably, the H3K4me3 enrichments at the *ifnb1*, *irf1*, *ifit1*, *ifit3*, *mx1*, *isg15*, and *ifi44l* TSSs were significantly increased in IVRPIE-overexpressing cells than those in control cells following IAV infection (Figure 4A). In contrast, the H3K27me3 levels at *ifnb1*, *irf1*, *ifit1*, *ifit3*, *mx1*, *isg15*, and *ifi44l* TSSs were impaired in infected IVRPIE-overexpressing cells than that in control cells (Figure 4B). Consistently, we observed a significant decrease in H3K4me3 levels and a significant increase in H3K27me3 levels at *ifnb1*, *irf1*, *ifit1*, *ifit3*, *mx1*, *isg15*, and *ifi44l* TSSs in knock down cells compared to control cells (Figures 4C,D). Taken together, these data suggest that IVRPIE may promote the IFN β1 and ISG transcription by affecting the histone modifications at the TSSs of these genes.

### hnRNP U Is Identified as an Interaction Partner for IVRPIE

**FIGURE 5:**
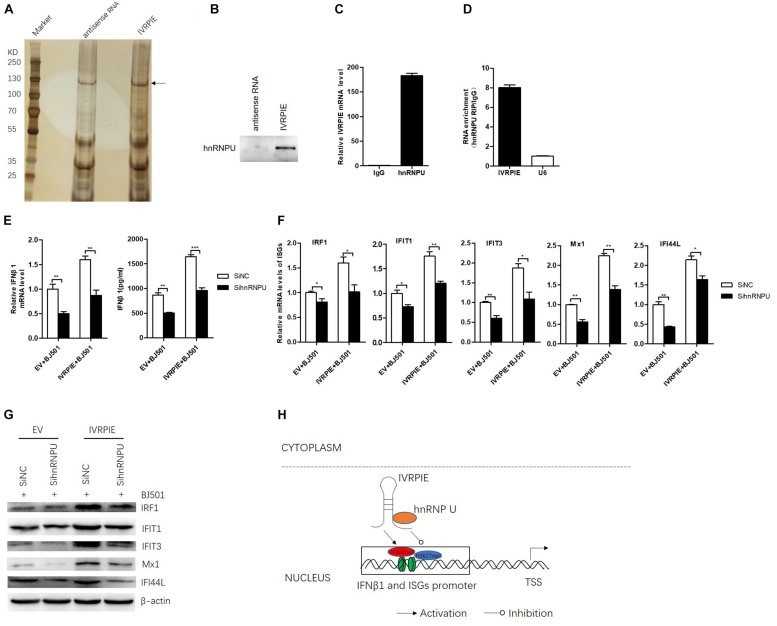
hnRNP U is identified as a protein partner of IVRPIE. **(A)** Silver staining of proteins pulled down by IVRPIE from A549 nuclear lysate. The whole bands were sent for mass spectrometry. **(B)** Western blotting confirms interaction between IVRPIE and hnRNP U *in vitro*. **(C,D)** hnRNP U RIP followed by RT-qPCR analysis of co-purified RNAs in A549 cells. IVRPIE level is significantly higher than that of control IgG antibody **(C)** and other nuclear RNAs normalized to IgG antibody **(D)**. **(E)** mRNA level and protein level of IFNβ1 were detected by RT-qPCR and ELISA respectively in hnRNP U knock-down IVRPIE overexpressing A549 cells and mRNA level of IFNβ1 was normalized to that of control cells. **(F,G)** mRNA levels and protein levels of ISGs were detected by RT-qPCR **(F)** and western blotting **(G)** in hnRNP U knock-down IVRPIE overexpressing A549 cells. **(H)** Schematic diagram of the mechanism for IVRPIE regulating IFNβ1 and ISG transcription.

To further understand the mechanism how IVRPIE affected the histone modifications of IFN β1 and ISGs, we performed RNA pull down assays to identify the protein partners of IVRPIE. To this end, we used *in vitro*-transcribed biotinylated IVRPIE or its antisense control RNA to pull down proteins from nuclear extracts of A549 cells. RNA-protein complexes were captured using streptavidin magnetic beads and subjected to SDS-PAGE and viewed by silver staining, and whole protein bands were sent for mass spectrometry. This approach identified several proteins that specifically bound with IVRPIE, one of which was identified to be hnRNP U (Figure 5A and Supplementary Table S4). We next confirmed the ability of hnRNP U to bind IVRPIE by western blotting (Figure 5B). RNA immunoprecipitation (RIP) was further performed to purify endogenous hnRNP U in A549 cells and qRT-PCR was used to analyze IVRPIE level. As predicted, the enrichment of IVRPIE in hnRNP U immunoprecipitates was significantly higher than that of control IgG antibodies (Figure 5C). Importantly, IVRPIE level in immunoprecipitates was also higher than other nuclear RNAs including U6 (Figure 5D). These results suggest that hnRNP U is a protein partner for IVRPIE’s antiviral function. We next explored the role of hnRNP U in IFNβ1, IRF1, IFIT1, IFIT3, Mx1, ISG15, and IFI44L expression. Specific siRNAs targeting hnRNP U were used to silence hnRNP U (Supplementary Figure S4A), and the IFN β1 and ISG mRNA levels were detected by RT-qPCR. Consistently, the siRNA-mediated silencing of hnRNP U led to significant decrease of IFN β1, IRF1, IFIT1, IFIT3, Mx1, and IFI44L expression in both IVRPIE overexpressing and natural cells (Figures 5E–G). These data indicate that hnRNP U is involved in IVRPIE regulation of IFN β1 and several ISG transcription (Figure 5H). However, we didn’t detect significant decrease of ISG15 expression, suggesting that there might be other mechanisms involved in regulation of IVRPIE in ISG expression.

## Discussion

Although antiviral signaling pathways in IAV infection have been well understood, the functions of non-coding RNA, especially lncRNA in these processes remain largely unclear. lncRNA NEAT, 7SL, NRAV, lnc-ISG20, and CCR5AS have been reported to regulate the innate immune responses to virus infection (Wang et al., 2007; Zhang et al., 2013; Ouyang et al., 2014; Chai et al., 2018; Kulkarni et al., 2019). In this study, we report a novel human lncRNA termed as IVRPIE, inhibits the IAV infection through promotion of the expression of IFNβ1 and several ISGs, such as IRF1, IFIT1, IFIT3, Mx1, ISG15, and IFI44L. hnRNP U was identified to be the protein partner for IVRPIE in this biological process. Our results further support the idea that lncRNAs play important roles in antiviral immune response.

As IFNβ stimulation did not promote the IVRPIE expression, we propose that IVRPIE is not one of the ISGs. Interestingly, some RNA viruses such as IAV, SeV, and VSV, or Poly (I:C) which is a TLR3 and MDA5 ligand, can induce IVRPIE while other RNA viruses such as RSV and DNA virus such as AdV cannot. We presume this may be related with different cellular receptors that different viruses bind to. Many candidate cellular receptors have been described for RSV entry, including annexin II, CX3 chemokine receptor 1 (CX3CR1), epidermal growth factor (EGF) receptor, calcium-dependent lectins, Toll-like receptor 4 (TLR4), intercellular adhesion molecule 1 (ICAM-1) receptors and heparan sulfate proteoglycans (HSPGs) (Griffiths et al., 2017). Recent studies have shown that IAV, SeV, and VSV are all recognized by RIG-I, and Poly (I:C) is recognized by TLR3 or MDA5, while AdV, as a DNA virus, is recognized by cGAS (Takeuchi and Akira, 2010). Thus, we presume that IVRPIE expression may be regulated by RIG-I, TLR3, MDA5, or the common downstream components shared by these pathways. However, our results showed that IVRPIE expression is not regulated by TBK1 or NF-κB. Further studies need to be done to investigate the mechanism involved in regulation of IVRPIE production. In this study, we found that the histone modifications (active mark H3K4me3 and repressive mark H3K27me3) at the TSSs of several ISGs were altered by IVRPIE, lending support to that lncRNAs regulate gene expression through chromatin remodeling. Since multiple mechanisms are involved in regulation of gene transcription, it may be worth investigating whether other mechanisms involved in regulation of IFNβ and ISG transcription.

We observed that IVRPIE altered the histone modifications of IFNβ1 and several ISGs through interaction with hnRNP U. It was reported that hnRNP U can inhibit HIV-1 gene expression by an N-terminal fragment specifically targeting the 3′ long terminal repeat (3′LTR) in the viral mRNA (Valente and Goff, 2006). In this study, hnRNP U exerted its antiviral function through a totally different way. In recent years, hnRNPs are identified as important functional partners for lncRNAs (Huarte et al., 2010; Carpenter et al., 2013; Atianand et al., 2016). A role for hnRNP U in the epigenetic regulation of gene expression by lncRNAs is also beginning to emerge (Hasegawa et al., 2010; Hacisuleyman et al., 2014). For example, hnRNP U is required for chromosomal localization of Xist RNA in the formation of the inactive X chromosome. hnRNP U is also required for cross-chromosomal co-localization of *Firre* in proper adipogenesis. In this study, hnRNP U was identified as a partner for IVRPIE, which adds further support to the role of hnRNPs in epigenetic regulation by lncRNAs. Secondary structure is important for molecular interaction between lncRNAs and proteins. Of course, further investigations need to be explored, such as the association between the sequence of lncRNA IVRPIE and secondary structures.

IVRPIE is located in the promoter region of TRAF family member associated NFκB activator (TANK). It has been reported that TANK is involved in antiviral activities by modulating NF-kappa B activation through interaction with TANK-binding kinase 1 (TBK1) or IKK epsilon (Chariot et al., 2002; Lee et al., 2013). Herein, our data indicated that TANK expression was not affected by IVRPIE and IVRPIE likely exerted antiviral function through non-TANK pathway. In addition, IVRPIE was mainly upregulated in peripheral blood immune cells and lung cells, not upregulated by other tissues, indicating that IVRPIE is mainly an antiviral regulator for blood and lung immune response.

## Data Availability Statement

Publicly available datasets were analyzed in this study. This data can be found here: GSE108807.

## Author Contributions

LZ contributed to the conception and design of the study, performed the experiments, analyzed the data, and the first draft of the manuscript. CL organized the database. MX, KW, and HF participated in the experiments. HG contributed to the conception and design of the study and provided important advice for experiment improvement. PY supervised the study, wrote sections of the manuscript, and revised it critically. XW supervised the study. PY and XW take primary responsibility for communication with the journal and editorial office during the submission process, throughout peer review and during publication.

## Conflict of Interest

The authors declare that the research was conducted in the absence of any commercial or financial relationships that could be construed as a potential conflict of interest.

## Footnotes

1 http://cpc2.cbi.pku.edu.cn

2 http://cpc2.cbi.pku.edu.cn

## Supplementary Material

FIGURE S1 Identification and characterization of IVRPIE. **(A)** RT-qPCR was used to validate the gene expression in RNA sequencing. **(B)** A549 cells were infected with several lncRNAs, and virus titers were detected using HA assay. **(C)** Protein-coding potential analysis was performed by coding potential calculator 2. **(D)** A549 cells were transfected with poly I:C (0.25 μg/mL) for indicated hours, and RT-qPCR was performed to determine the IVRPIE expression. **(E)** A549 cells were stimulated with IFNβ for indicated hours, and RT-qPCR was performed to determine the IVRPIE expression. **(F)** BEAS-2B cells were infected with different viruses, and RT-qPCR was performed to determine the IVRPIE expression. **(G)** BEAS-2B cells were transfected with poly I:C (2μg/mL) for 16 h or stimulated with IFNβ for 24 h, and RT-qPCR was performed to determine the IVRPIE expression. RT-qPCR was performed to determine the IVRPIE expression. **(H)** Different cell lines were infected with BJ501, and RT-qPCR was performed to determine the IVRPIE expression. **(I)** A549 cells were pretreated with DMSO, MRT67307 HCl, or Pyrrolidinedithiocarbamate ammonium, followed by BJ501 infection for 24 h. RT-qPCR was performed to determine the IVRPIE expression. Data were normalized to GAPDH. Data are shown as the mean ± SD; *n* = 3.

FIGURE S2 Altering IVRPIE expression regulates IAV or VSV replication. **(A)** RT-PCR was used to amplify IVRPIE. **(B)** IVRPIE was overexpressed in A549 cells and mRNA level was detected by RT-qPCR. **(C)** IVRPIE was silenced by specific ASOs in A549 cells and mRNA level was detected by RT-qPCR. **(D,E)** IVRPIE was transiently overexpressed **(D)** or specifically knocked down **(E)** in BEAS-2B cells, and viral hemagglutinin (HA) was detected by western blotting. **(F,G)** A549 cells were transfected with pcDNA 3.1-IVRPIE **(F)** or specific ASOs targeting IVRPIE **(G)**, followed by VSNJV infection (100μL, 5 × 10^7^ TCID_50_/mL) for 24 h, and virus titers were determined using TCID_50_ assay. Data were normalized to GAPDH. Data are shown as the mean ± SD; *n* = 3. **P* < 0.05; ***P* < 0.01; ****P* < 0.001 (Student’s *t*-test).

FIGURE S3 Regulation of TANK expression by IVRPIE. **(A,B)** IVRPIE was overexpressed **(A)** or knocked down **(B)** in A549 cells, and RT-qPCR was used to detect TANK expression. Data were normalized to GAPDH. Data are shown as the mean ± SD; *n* = 3.

FIGURE S4 Knock-down of hnRNP Uin A549 cells. **(A)** hnRNP U was knock down in A549 cells, and RT-qPCR was used to detect hnRNP U expression. Data were normalized to GAPDH. Data are shown as the mean ± SD; *n* = 3. **P* < 0.05; ***P* < 0.01; ****P* < 0.001 (Student’s *t*-test).

TABLE S1 Primers used for RT-PCR, RT-qPCR and ChIP-qPCR.

TABLE S2 lncRNAs Differentially Expressed in Patients with IAV Infection.

TABLE S3 Exact sequence of IVRPIE.

TABLE S4 Top 10 Proteins Pulled Down by IVRPIE.
