# Supplementary figures and images for: A Long Non-coding RNA IVRPIE Promotes Host Antiviral Immune Responses Through Regulating Interferon β1 and ISG Expression
Source: Front Microbiol. 2020 Feb 20;11:260. doi: 10.3389/fmicb.2020.00260 (PMC7044153; doi:10.3389/fmicb.2020.00260)

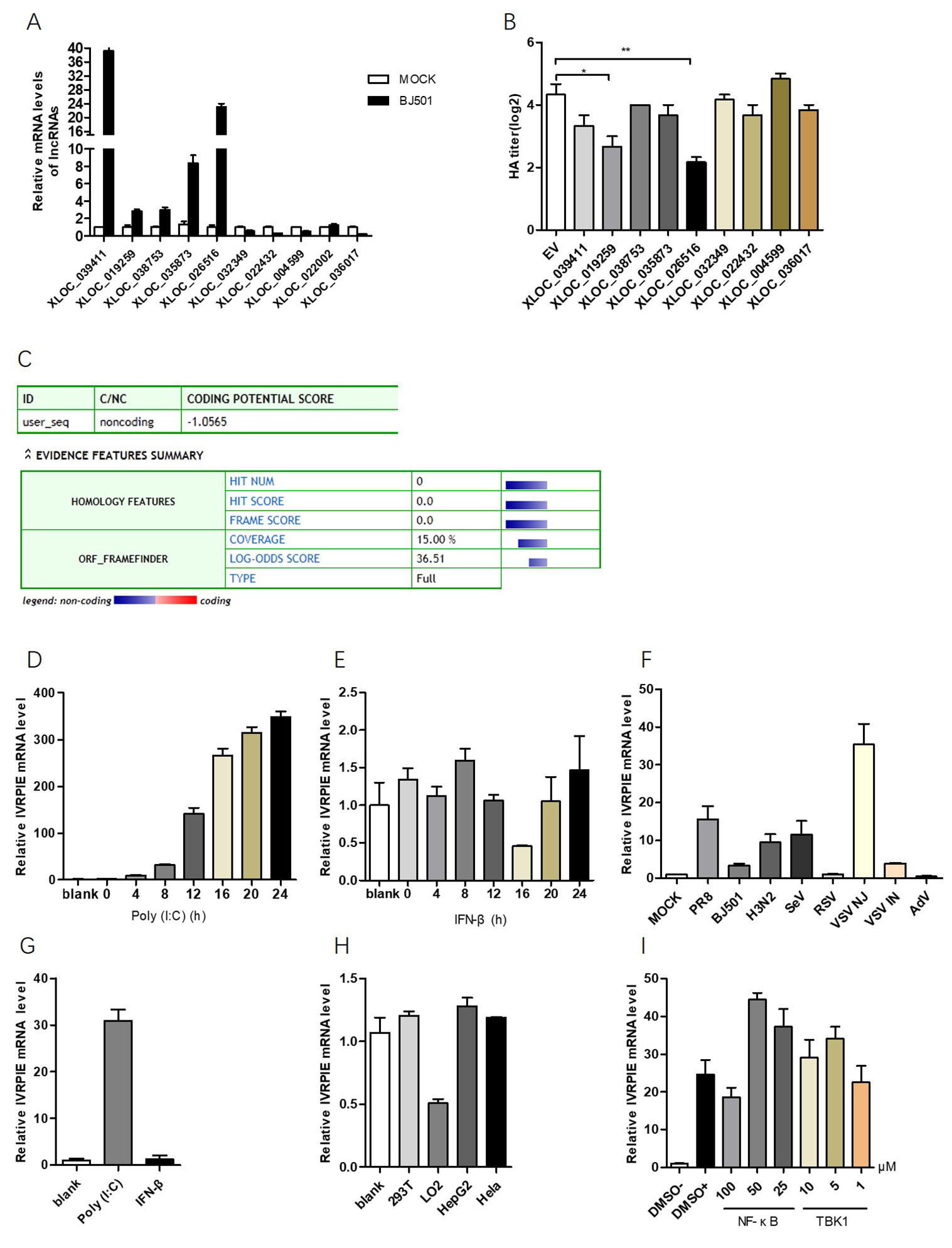

Supplement: FIGURE S1 — Identification and characterization of IVRPIE. (A) RT-qPCR was used to validate the gene expression in RNA sequencing. (B) A549 cells were infected with several lncRNAs, and virus titers were detected using HA assay. (C) Protein-coding potential analysis was performed by coding potential calculator 2. (D) A549 cells were transfected with poly I:C (0.25 μg/mL) for indicated hours, and RT-qPCR was performed to determine the IVRPIE expression. (E) A549 cells were stimulated with IFNβ for indicated hours, and RT-qPCR was performed to determine the IVRPIE expression. (F) BEAS-2B cells were infected with different viruses, and RT-qPCR was performed to determine the IVRPIE expression. (G) BEAS-2B cells were transfected with poly I:C (2μg/mL) for 16 h or stimulated with IFNβ for 24 h, and RT-qPCR was performed to determine the IVRPIE expression. RT-qPCR was performed to determine the IVRPIE expression. (H) Different cell lines were infected with BJ501, and RT-qPCR was performed to determine the IVRPIE expression. (I) A549 cells were pretreated with DMSO, MRT67307 HCl, or Pyrrolidinedithiocarbamate ammonium, followed by BJ501 infection for 24 h. RT-qPCR was performed to determine the IVRPIE expression. Data were normalized to GAPDH. Data are shown as the mean ± SD; n = 3. [file Image_1.JPEG]

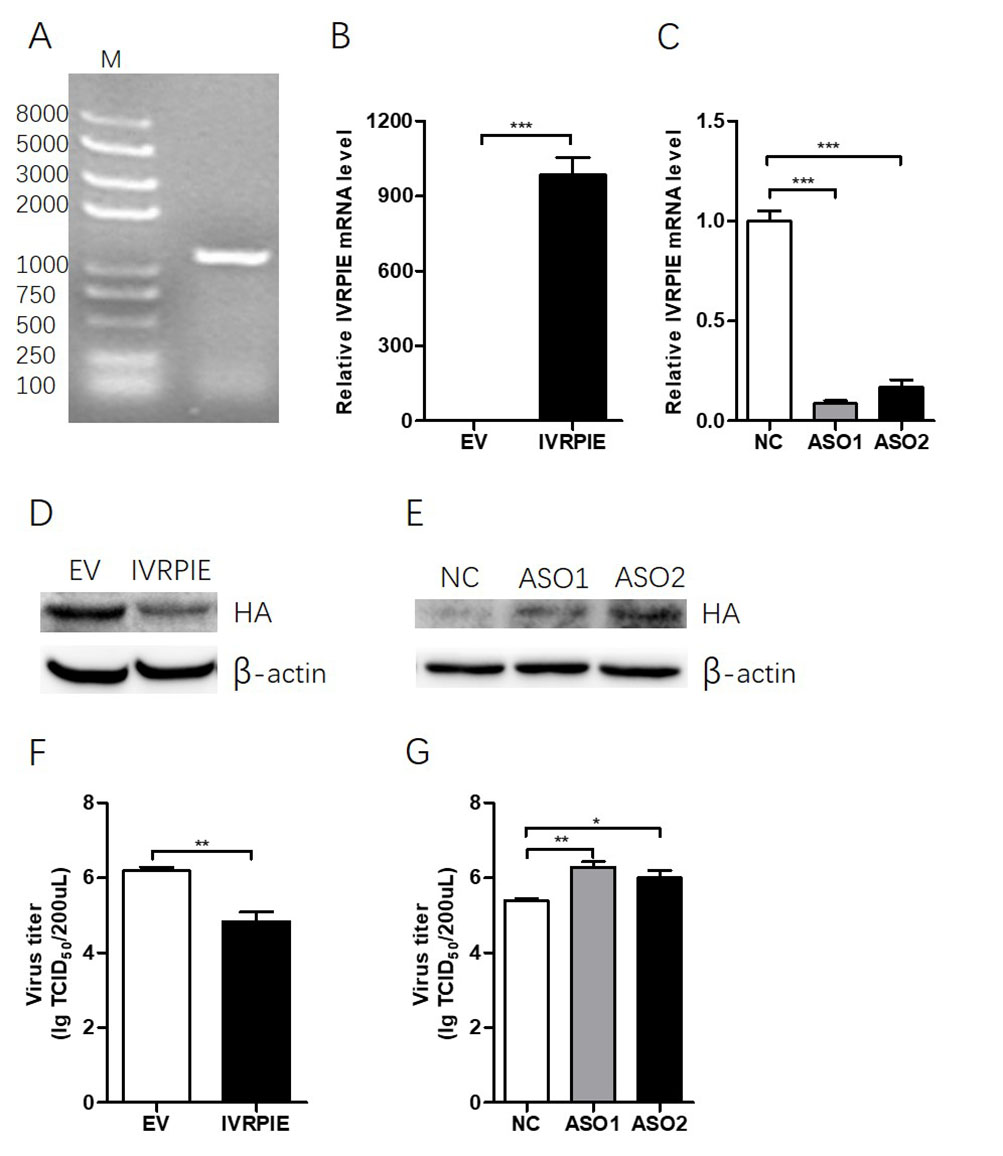

Supplement: FIGURE S2 — Altering IVRPIE expression regulates IAV or VSV replication. (A) RT-PCR was used to amplify IVRPIE. (B) IVRPIE was overexpressed in A549 cells and mRNA level was detected by RT-qPCR. (C) IVRPIE was silenced by specific ASOs in A549 cells and mRNA level was detected by RT-qPCR. (D,E) IVRPIE was transiently overexpressed (D) or specifically knocked down (E) in BEAS-2B cells, and viral hemagglutinin (HA) was detected by western blotting. (F,G) A549 cells were transfected with pcDNA 3.1-IVRPIE (F) or specific ASOs targeting IVRPIE (G), followed by VSNJV infection (100μL, 5 × 10^7^ TCID50/mL) for 24 h, and virus titers were determined using TCID50 assay. Data were normalized to GAPDH. Data are shown as the mean ± SD; n = 3. *P < 0.05; **P < 0.01; ***P < 0.001 (Student’s t-test). [file Image_2.JPEG]

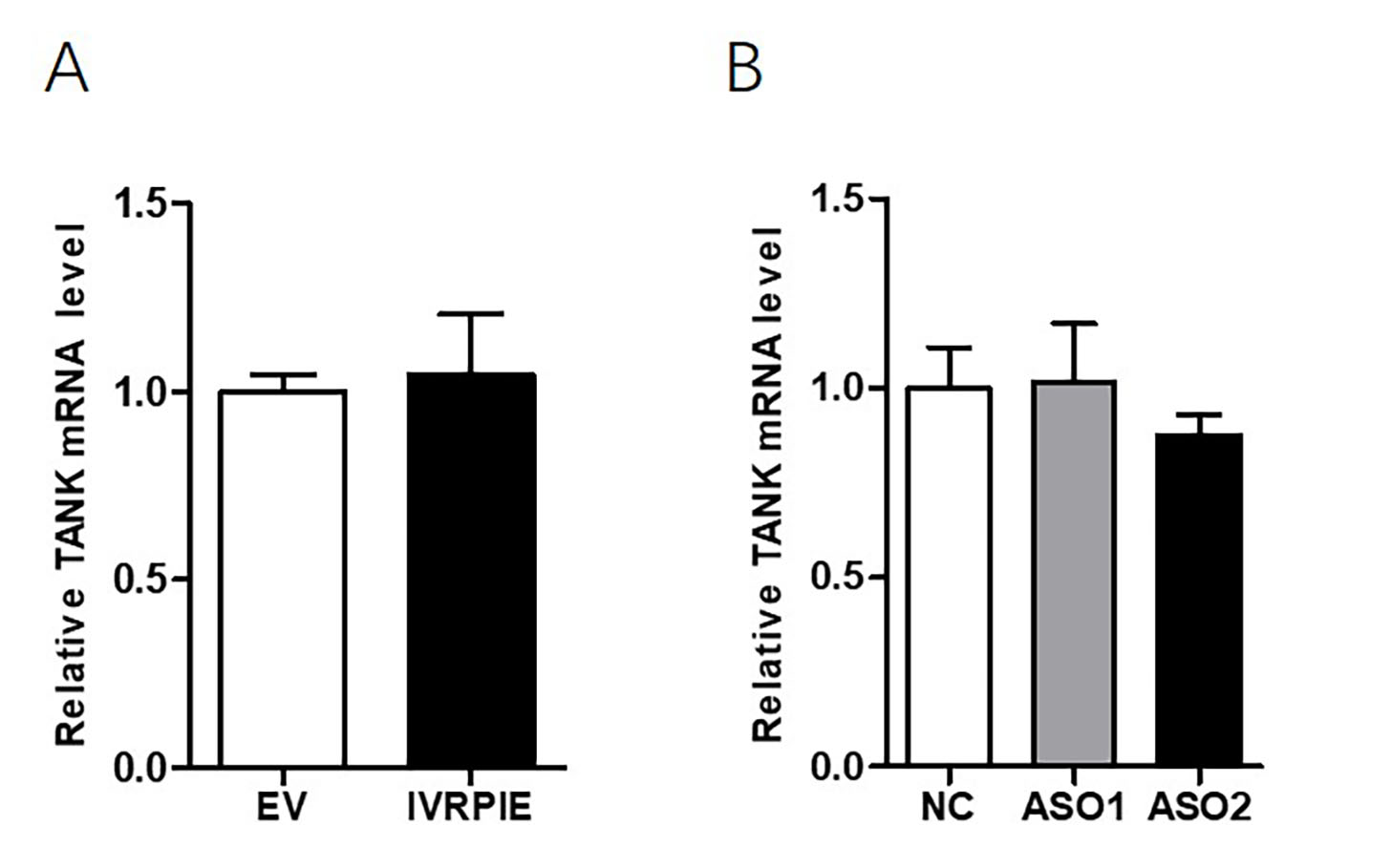

Supplement: FIGURE S3 — Regulation of TANK expression by IVRPIE. (A,B) IVRPIE was overexpressed (A) or knocked down (B) in A549 cells, and RT-qPCR was used to detect TANK expression. Data were normalized to GAPDH. Data are shown as the mean ± SD; n = 3. [file Image_3.JPEG]

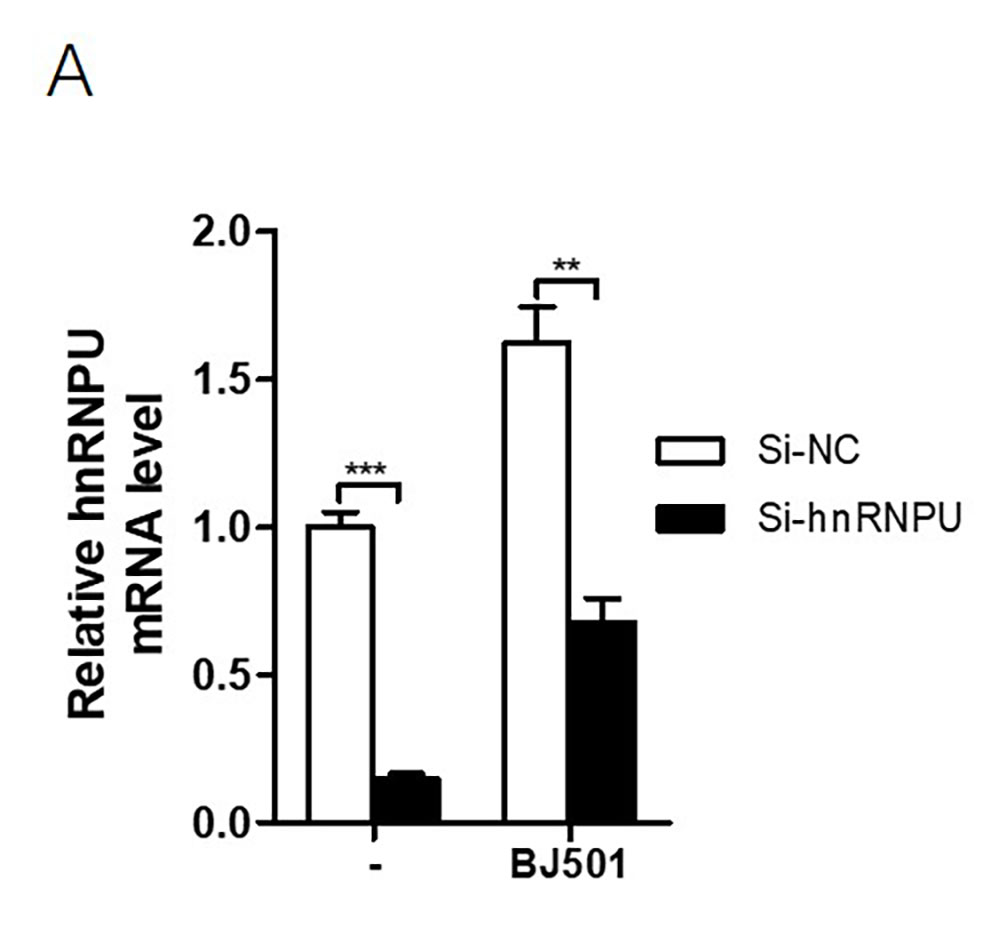

Supplement: FIGURE S4 — Knock-down of hnRNP Uin A549 cells. (A) hnRNP U was knock down in A549 cells, and RT-qPCR was used to detect hnRNP U expression. Data were normalized to GAPDH. Data are shown as the mean ± SD; n = 3. *P < 0.05; **P < 0.01; ***P < 0.001 (Student’s t-test). [file Image_4.JPEG]
