# Supplementary material for: A Long Non-coding RNA IVRPIE Promotes Host Antiviral Immune Responses Through Regulating Interferon β1 and ISG Expression
Source: Front Microbiol. 2020 Feb 20;11:260. doi: 10.3389/fmicb.2020.00260 (PMC7044153; doi:10.3389/fmicb.2020.00260)
Supplement: TABLE S3 — Exact sequence of IVRPIE. [file Table_3.docx]

| IVRPIE |
| --- |
| CTCGTGACTGTAGCTCTGACTTCCTTTTGAAGCAAGATACTTTATGTGAATAGTTAAGGCATTTTAACAACAACAACAACAACAAAAACCCCAGGAATTGTAAACACAAAAATACTTTTGGATTAAGCATTTTATGTAAGAAAATTTAAAAGTTCAGTTTATTTGATAAGTGGCTTTTTAACCTTTAAGAAGTCGCTTTTTGACCTAAGAAGCATGTTTATAAACAGGAGGTAAAAACCAAATTTGATTATAGTTGTCTTTATGGCCTACAGAAGTCACTTGGAAGAAGCTTTGTAGTAATAAGGTTTAAGCTCAATTGCAGAGCCTGAGCTTAGGTACTCAGAAGAGAGGTGAAGCAAACAAGGCTCTTAAGACAGCTTTTCTGCCTTGAGAACTTAAGGTTCACTGTTCGTGGGATAAAAGTGTGTTCCAGCAAACTCGGGAAATGCTTTACAAATACATGTATTTCTCTTAGGTTGCTTAGAGCAAAACATCACATTTTACATGCAAACGGAAGACAGCATGTGCACCACAGACATTTTTCTTTGATATAAAATTTTAGAGCTGGAAATGTCCTTAGACAACATCTGTTCCATTTCCTTTTTATTACAGATGAGGGAAACAGGAGAAGGTAAATGCTCAATGTCACAAGTAAAGTTAGAAGCAGCGCTTAGATTAAAATGTGAGTCTTTTGAATCCAAGTGCATTTCTTTTTTTTACTGCACTACACTCTTGGCATATAAAGTTACTGAGCTGGATAAAAACTGAATTAAGCAGTCTCCTTTTTCAGCTCCTGCATTTTACAGATGAGAAGTCTGAGGCTTTACCTAAACTTCAACAGTTATTAAAAGGTCTAGAATTTAGAACTTCTGATTCTTAGTCTAGTTTGTTTTCCACTTTGAAACACTGCCCTCTCTTAAATTATATGTTAAATAAATCTGAGATATTGCTACTTATGATGAAATTATTCTATAAAAAATAATTTCAAAAGTATCTAAAATGCTGAATAAACTTATGATTAAAGGCTTCTACGTACTCATCTAACATACAATGAAGCATTACATTTCTTTTGGAACTATGGGTTTTATAACTATCTTACTGATGAAATGCTAAATATTAAATGCTATTGTGTGCAATGGAAAAATAAAACCAACCTATCCCGTGCTGTAAATATAGCAAAACAAACCAACAAAAAAGTGAGATAGCCACTAGTGAAAGGAAAACTTACTTCTTAAGAATTTACAGTTTTGAATGAGCAGAGTTTTAAAAACACAGCTTTTAAAAAAAGCAGCGCTCCTGTCTAGACTAAAAAAAAA |

Table S3. Exact sequence of IVRPIE
